# Health Information Technology Continues to Show Positive Effect on Medical Outcomes: Systematic Review

**DOI:** 10.2196/jmir.8793

**Published:** 2018-02-05

**Authors:** Clemens Scott Kruse, Amanda Beane

**Affiliations:** ^1^ School of Health Administration Texas State University San Marcos, TX United States

**Keywords:** health information technology, treatment outcome, electronic health record

## Abstract

**Background:**

Health information technology (HIT) has been introduced into the health care industry since the 1960s when mainframes assisted with financial transactions, but questions remained about HIT’s contribution to medical outcomes. Several systematic reviews since the 1990s have focused on this relationship. This review updates the literature.

**Objective:**

The purpose of this review was to analyze the current literature for the impact of HIT on medical outcomes. We hypothesized that there is a positive association between the adoption of HIT and medical outcomes.

**Methods:**

We queried the Cumulative Index of Nursing and Allied Health Literature (CINAHL) and Medical Literature Analysis and Retrieval System Online (MEDLINE) by PubMed databases for peer-reviewed publications in the last 5 years that defined an HIT intervention and an effect on medical outcomes in terms of efficiency or effectiveness. We structured the review from the Primary Reporting Items for Systematic Reviews and Meta-Analysis (PRISMA), and we conducted the review in accordance with the Assessment for Multiple Systematic Reviews (AMSTAR).

**Results:**

We narrowed our search from 3636 papers to 37 for final analysis. At least one improved medical outcome as a result of HIT adoption was identified in 81% (25/37) of research studies that met inclusion criteria, thus strongly supporting our hypothesis. No statistical difference in outcomes was identified as a result of HIT in 19% of included studies. Twelve categories of HIT and three categories of outcomes occurred 38 and 65 times, respectively.

**Conclusions:**

A strong majority of the literature shows positive effects of HIT on the effectiveness of medical outcomes, which positively supports efforts that prepare for stage 3 of meaningful use. This aligns with previous reviews in other time frames.

## Introduction

### Background

Health information technology (HIT) is an umbrella term that covers a wide range of technologies that store, share, and analyze health information [1,2]. In this role, HIT can influence service quality and provider performance [3]. As stated by Wagner et al, the adoption of HIT for the purpose of improving medical outcomes was touted by the Institute of Medicine in numerous reports, including “The Computer-based Patient Records: An Essential Technology for Health Care” in 1991, “To Err is Human: Building a Safer Health Care System” in 2002, and “Patient Safety: Achieving a New Standard for Care” in 2004 [4]. Due to the costs associated with implementing HIT, initially, health care payers seemed to be the only stakeholders benefiting from it [4].

In the United States in 2009, the Health Information Technology for Economic and Clinical Health (HITECH) Act made incentive payments available to providers who adopted the *meaningful use* of a certified electronic health record (EHR), stimulating widespread adoption of HIT across various health care settings [4]. Since the implementation of the HITECH Act, adoption rates of the electronic medical record in the United States have shown greater than arithmetic growth [5], but have patients experienced a commensurate increase in quality and decrease in errors as a result of the presence of that technology? The same question applies internationally. In 2014, Denmark instituted a regional patient record system. In 2015, Switzerland adopted a nationwide EHR, and Germany issued chip-based medical cards to all statutory health insurance beneficiaries for health care claims. In 2018, Germany will penalize providers who do not participate fully in this program. Have outcomes followed these programs?

Over the last several years, many studies have examined a relationship between the use of HIT and resulting health outcomes, administrative efficiencies, and cost [1,2]. Most studies that we found after our initial interest demonstrated a positive relationship between the use of HIT and medical outcomes, and these studies spanned seven countries [6-12]. However, all but three lacked quality metrics [1,2,11]. Three previous studies reviewed relative literature published in various but distinct time frames from 1995 and 2010.

Buntin et al [1] evaluated the benefits of health information technology in 2011 using data available from 2007 to 2010. This was a continuation of effort from Chaudhry et al, who examined literature from 1995 to 2004 and Goldzweig et al, who examined literature from 2004 to 2007 [2,11]. These three reviews demonstrated higher standards of science in their analysis, and therefore, this review will examine the years from January 1, 2011 to July 31, 2017 to update the literature. A good question to ask, however, is how has this changed since the HITECH Act? What has been the result of medical outcomes, specifically, since the last high quality review was conducted [1]?

### Objective

The purpose of this review was to evaluate the current literature demonstrating the impact of HIT adoption on medical outcomes. Using the same methods as Buntin et al, Chaudhry et al, and Goldzweig et al (2004, 2007, and 2011, respectively), we intended to carry this research forward into 2017 [1]. What is the effect of the adoption of health information technology on medical outcomes since 2011? The hypotheses are as follows:

Hypothesis 1: There is a positive association between the adoption of HIT and medical outcomes.

Hypothesis 0: There is no positive association between the adoption of HIT and medical outcomes.

## Methods

### Eligibility Criteria

The conduct of our review followed a measurement tool for the “Assessment of Multiple Systematic Reviews” (AMSTAR) [13]. The format of the review follows the Preferred Reporting Items for Systematic Reviews and Meta-Analyses (PRISMA) [14]. The search criteria matched that used by Chaudhry, Goldzweig, and Buntin and colleagues. Papers were eligible for selection in this systematic review if they were published in the last 5 years in academic (peer-reviewed) journals, in English, whose full-text was available for analysis, and the papers addressed implementation of HIT and an association with an effect on medical outcomes expressed in terms of efficiency or effectiveness. We chose 5 years because that is the amount of time since the last review was published on this topic. We limited the search to peer-reviewed journals to ensure an acceptable element of quality to the papers we were analyzing. We made the decision not to include other systematic reviews in the analysis, but they were used in the Discussion section for comparison.

### Information Sources

We queried two common research databases: Medical Literature Analysis and Retrieval System Online (MEDLINE) by PubMed and the Cumulative Index of Nursing and Allied Health Literature (CINAHL). We used key terms from the US National Library of medicine’s medical subject headings separated by Boolean terms. Searches were conducted from July 1, 2017 to July 4, 2017.

### Search and Study Selection

Searches in each database were nearly identical. Due to the differences in indexing methods between the databases, we had to slightly modify the search string and filters for each. We screened for date of publication to begin in 2007 until the end of June 2017. The filters in PubMed enabled us to screen out reviews. In CINAHL, we excluded MEDLINE because it was being collected separately from PubMed, and this eliminated most duplicates. Papers were placed into an Excel (Microsoft) spreadsheet shared among the reviewers. Remaining duplicates were removed. As a quality measure, only peer-reviewed journals were used in the selection process.

### Data Collection Process and Data Items

Reviewers agreed ahead of time what to look for in each abstract. We focused on papers that described a technological intervention that follows the definition of previous reviews [1,2,11] and that expressed medical outcomes in terms of either effectiveness or efficiency. After the initial search was completed, we removed duplicates and filtered. Each member of the review team read all of the remaining abstracts to ensure they were reviewed at least twice, as outlined by AMSTAR [15]. Independent notes were taken on a shared spreadsheet to inspire discussion. Two consensus meetings were held: one to identify the full-text papers for analysis and one to identify other observations for additional analysis. A statistic of agreement, *kappa*, was calculated.

### Summary Measures, Synthesis of Results, and Bias

The summary measure used in this analysis was the medical outcome specified in terms of either efficiency or effectiveness. When clear statistics were listed, our team recorded them for our analysis. We also identified signs of bias that could have deleterious effect on the broad application of the results. Several papers only mentioned advantages of administrative efficiency, such as a shorter length of stay (LOS) and lower readmission rates. These were kept because, we reasoned, a shorter LOS could have been due to improved outcomes, and lower readmission rates could have been enabled with improved outcomes that would have otherwise caused the patient to return.

## Results

### Eligibility Criteria, Information Sources, and Search and Study Selection

Studies from PubMed and CINAHL that defined an HIT intervention and a corresponding effect on medical outcomes stated in terms of efficiency or effectiveness were eligible for selection. The search for this review was extensive, and the reviewers took care to be deliberate and thorough in their process.

### Data Collection Process and Data Items

**Figure 1:**
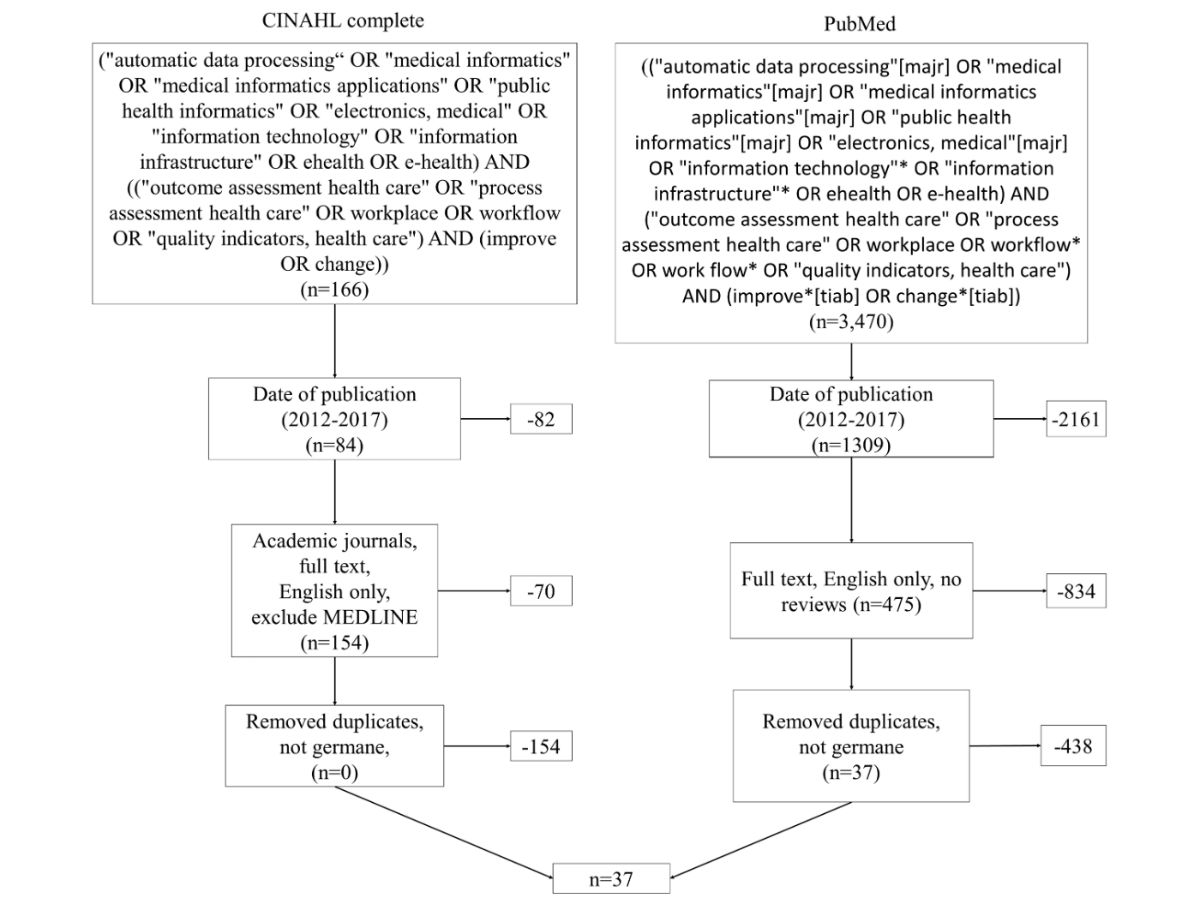
Literature search with inclusion and exclusion criteria.

The initial search, as illustrated in Figure 1, resulted in 3636 results. After removing duplicates and filtering, the remaining 629 abstracts were read in their entirety by the two reviewers, as outlined by AMSTAR [15]. Independent notes were taken on a shared spreadsheet. After the first consensus meeting, 534 were eliminated because they did not report medical outcomes, 8 because they were editorials, 8 were protocols and reported no results, 6 were models without results, and 6 were not germane to the objective. A statistic of agreement, *kappa*, was calculated to be .966, which is indicative of a high level of agreement. Only 54 studies remained for full-text analysis, although some were kept under suspicion because the abstract was vague on whether or not outcomes were reported. A similar review approach was used for the analysis of the full-text papers. After the second consensus meeting, 15 more were removed for no medical outcomes and 2 removed for not being germane to the objective. The final set for analysis was 37.

### Summary Measures, Synthesis of Results, and Bias

Multimedia Appendix 1 summarizes the results of the analysis of the 37 studies chosen. It lists the descriptive title of the study, the HIT intervention, the measures of efficiency or effectiveness, and any bias observed that could limit the applicability of the results [15-51].

After consensus meeting number two, the categories of HIT recorded by each reviewer were combined. We counted the number of times that a category occurred in the literature and sorted by frequency of occurrence. This data was placed into an affinity matrix for further analysis (see Table 1).

**Table 1:** The specific categories of health information technology (HIT) and their frequency of occurrence.

Category of HIT^a^	Paper in which category occurred	Frequency (n=38)
Web-based	[19,31,34,41,44,45,47,51]	8
Telemedicine	[16-18,21,32,40,46]	7
Software	[25,27,28,30,36,50]	6
CDSS^b^	[22,24,33,35,48]	5
mHealth^c^	[20,42,45]	3
Telemonitoring	[29,37]	2
Electronic ordering	[26,38]	2
HIT	[49]	1
HIE^d^	[43]	1
Robot assist	[15]	1
Videoconferencing	[23]	1
Remote screening	[39]	1

^a^HIT: health information technology.

^b^CDSS: clinical decision support systems.

^c^mHealth: mobile health.

^d^HIE: health information exchange.

**Table 2:** The specific categories of outcomes and their frequency of occurrence.

Category of outcome	Paper in which category occurred^a^	Frequency
Physical	[15-17,23-26,28,30-35,37,39,41,42,44,45,48,49,51]	39
Psychological	[17-19,21,27,40,47]	13
Continuity of care	[20,22,23,25,27,29,36,39,40,43,46,50]	13
Total	Multiple occurrences in same paper	65

^a^More than one occurrence was observed in the following papers in the categories of outcome; physical: 15-17, 23, 28, 37, 39, 41, 42; psychological: 18, 21, 27, 40; continuity of care: 23.

Twelve different categories of HIT were identified in our analysis with a total of 38 occurrences. Web-based interventions were analyzed most frequently at 8 of 38 occurrences (21%) [19,31,34,41,44,45,47,51]. Telemedicine and software programs were the next most frequently identified interventions, occurring 7 of 38 occurrences (18%) [16-18,21,32,40,46] and 6 of 38 occurrences (16%) [25,27,28,30,36,50], respectively. Clinical decision support systems were analyzed at a frequency of 5 of 38 occurrences (13%) [22,24,33,35,48]. mHealth occurred 3 of 38 occurrences (8%) [20,42,45]. Telemedicine [29,37] and electronic ordering [26,38] HIT interventions occurred 2 of 38 occurrences (5%). Health information exchange (HIE) [43], HIT [49], robot assistance [15], videoconferencing [23], and remote screening [39] were all identified once for the lowest frequency at 3% individually.

Table 2 tabulates the categories of outcomes and their frequency of occurrence.

The asterisks note that more than one occurrence was observed within the same paper. Evidence of efficiency or effectiveness that were grouped under physical outcomes included body mass index, blood pressure, hypertension, pain, infection, activities of daily living, mortality, vaccines nutrition, physical activity, cardiovascular disease, wound healing, diabetes distress, quality of life, A1C level, low-density lipoprotein, vaccination rate, sedation interruptions, spontaneous breathing trials, mechanical ventilations, asthma control, cholesterol, and cluster of differentiation 4 count. Occurrences grouped as psychological included depression, insomnia, self-efficacy, emotional stability, maintenance of motivation, upset, negative mood states, social outcomes, and eating disorder symptomatology. Continuity of care included medication administration, medication adherence, service utilization, readmission, length of stay, unmet needs, and reduced office visits. Although readmission, length of stay, and reduced utilization are qualities most often associated with administrative efficiencies, we chose to keep these in the review because these efficiencies could have been enabled because of improved medical outcomes. The most common outcome category was physical, which appeared 39 of 65 occurrences (60%) [15-17,23-26,28,30-35,37,39,41,42,44,45,48,49,51]. The other two categories tied for second most often were as follows: psychological [17-19,21,27,40,47] and continuity of care [20,22,23,25,27,29,36,39,40,43,46,50], which each occurrences 13 of 65 occurrences (20%). Table 3 illustrates the categories of outcomes and the outcome results and their frequency of occurrence.

**Table 3:** Outcome results and their frequency of occurrence.

Result of outcome	Paper in which result occurred	Frequency (n=37)
Improved	[18,20-29,31-35,37-46,48-51]	30
No statistical difference	[15-17,19,30,36,47]	7

Of the 37 papers included, 30 (81%) reported an improvement in efficiency or effectiveness related to a medical outcome. No statistical difference in outcomes was reported in 7 of 37 occurrences (19%) studies. These results strongly support our hypothesis; therefore, we accept our hypothesis and reject the null. There is a positive association between the adoption of HIT and medical outcomes.

We made 15 comments related to bias in the original research, the majority of which were related to the lack of heterogeneity in characteristics of participants. Characteristics that were noted include socioeconomic status, gender, age, ethnicity, and geographical area. Small sample size was noted as a potential concern in 4 studies, and participation refusal or dropout was noted in 2 studies. In one study, participants received payment for participation, and in another study, two of the authors had invented the technology being evaluated. Other sources of bias identified included outcomes reported based on a quality manager’s response to survey, seasonal influences not controlled for, and technical challenges experienced that resulted in delays.

## Discussion

### Summary of Evidence

Health care providers will continue to be incentivized to adopt HIT as policy makers respond to quality, and safety concerns and reimbursement methods transition toward value-based purchasing [4]. Providers, consumers, and policy makers alike stand to benefit from the further proliferation of HIT. Our research aligns with previous work that identified improvements achieved as the result of the adoption of HIT [1,2,11]. The majority of research we identified, 81%, in this review demonstrated improved medical outcomes in terms of efficiency or effectiveness as a result of HIT adoption. Although these findings are similar to the overall conclusions drawn by previous reviews, the adoption of HIT can have a positive impact on medical outcomes [1,2,11]. There are two key differences between our work and the three previous literature reviews.

First, Buntin et al reported that less than 10% of the studies included in their work demonstrated negative findings related to the adoption of HIT [1]. We identified a number of studies that demonstrated no statistical improvement, but we did not identify any negative impacts as a result of the adoption of HIT. Buntin et al noted that the majority of their negative findings were associated with provider satisfaction with HIT. We chose to only include papers that demonstrated effects of efficiency and effectiveness in terms of medical outcomes; this could account for the difference in our findings. Organizational factors related to the success of HIT implementation and improved medical outcomes is one area where further research is needed [1,2,11].

Second, the literature review conducted by Chaudhry et al in 2004 noted that the improved outcomes demonstrated were reported by a limited set of large benchmark organizations and cautioned on the ability to generalize positive findings to other institutions [2]. Goldzweig et al and Buntin and colleagues identified the emergence of more widespread research outside larger and more established organizations [11,1]. One important finding noted by Goldzweig et al was an increased focus on patient-focused HIT. We believe this trend has continued through 2017. We identified a greater variety in the types of HIT being studied than previous literature reviews; Web-based interventions being the most frequently researched. This may be an indication of an increased rate of adoption of HIT and perhaps improved efficiency and effectiveness across a wider variety of health care settings.

One common theme in all four literature reviews is the limited amount of research associated with HIE specifically [1,2,11]. HIE is at the forefront of technological advancement in the health care industry [4]. Only one study in our review of recent literature included HIE. More research is needed to identify the outcomes associated with the adoption of HIT systems that are capable of information exchange.

### Limitations

Our literature review did not identify any studies demonstrating a negative impact on medical outcomes as the result of HIT adoption. The absence of negative findings may be because of publication bias [1] and should be considered in the interpretation of these results. This is supported by the finding of 19% of studies that found no statistical difference in outcomes as a result of HIT. Another limitation of this work is the diversity in types of medical outcomes examined and the uniqueness of each sample studied. This impacts the ability to generalize findings across the industry. Furthermore, limiting our search to MEDLINE by PubMed and CINAHL may have impacted the scope of our results.

### Conclusions

HIT has the potential to improve the quality and safety of health care services. Providers who leverage HIT to improve medical outcomes can position themselves for sustainability in the future. Further research is needed to continue to reveal and define the relationship between the adoption of HIT and medical outcomes. This will be especially true as the industry establishes new and innovative ways to integrate technological advances and works toward greater interoperability as the United States prepares for stage 3 of meaningful use, as all providers seek a link between the application of HIT in health care and its effect on outcomes, and as other nations such as Switzerland, Denmark, and Germany reconcile national medical programs such as a nationwide EHR, regional electronic patient record system, and national medical chip cards, respectively, against outcomes.

## Appendix

Multimedia Appendix 1 Summary of analysis.

## Abbreviations

AMSTAR: Assessment for Multiple Systematic Reviews
CINAHL: Cumulative Index of Nursing and Allied Health Literature
EHR: electronic health record
HIE: health information exchange
HIT: health information technology
HITECH: Health Information Technology for Economic and Clinical Health
LOS: length of stay
MEDLINE: Medical Literature Analysis and Retrieval System Online
mHealth: mobile health
PRISMA: Primary Reporting Items for Systematic Reviews and Meta-Analysis
