# Supplementary material for: Health Information Technology Continues to Show Positive Effect on Medical Outcomes: Systematic Review
Source: J Med Internet Res. 2018 Feb 5;20(2):e41. doi: 10.2196/jmir.8793 (PMC5818676; doi:10.2196/jmir.8793)
Supplement: Multimedia Appendix 1 [file jmir_v20i2e41_app1.pdf]

Appendix 1: Summary of analysis

Study	Technology intervention	Effectiveness or efficiency	Bias and quality measures
A randomized trial of telemedicine efficacy and safety for nonacute headaches [15]	One-time telemedicine consultations for nonacute headache	Health characteristics of all participants were collected for both the control and intervention group: body mass index, neck pain, insomnia, and hypertension. Although there were slight changes for both groups in these characteristics, there was no significant difference between them.	No bias detected<br>Randomized noninferiority trial telemedicine group (N=200), traditional (N=202)
Results from a clinical yoga program for veterans: yoga via telehealth provides comparable satisfaction and health improvements to in-person yoga [16]	telehealth (yoga)	More than 80% of participants who endorsed a problem with pain, energy level, depression, or anxiety reported improvement in these symptoms. Those who participated via telehealth did not differ from those who participated in-person in any measure of satisfaction, overall improvement (*P*=.40), or improvement in any of 16 specific health problems: back pain, other pain, headaches, upset stomach, constipation or diarrhea, trouble falling or staying asleep, energy level, irritable, angry outbursts, depression, difficulty	Participants were predominantly male.<br>True experiment, control (N=29), intervention (N=30)

		concentrating, anxiety, easily startled, repeated disturbing memories, and other.	
Comparison of robotic and laparoscopic colorectal resections with respect to 30-day perioperative morbidity [17]	Robot-assisted surgery for laparoscopic colorectal resections	No difference in operative duration or requirement for blood transfusion. Robotic method had decreased incidence of unplanned conversion to open surgery compared with laparoscopic. No difference in postoperative outcomes of postoperative ileus, anastomotic leak, venous thromboembolism, wound infection, cardiac complications, and pulmonary complications. No difference in operative duration or requirement for blood transfusion. Robotic method had decreased incidence of unplanned conversion to open surgery compared with laparoscopic.	No bias identified. Quasi-experimental (nonrandomized), control (N=7920), intervention (N=472).
Adherence and factors affecting satisfaction in long-term telerehabilitation for patients with	Telemedicine for rehabilitation of chronic obstructive pulmonary disease (COPD) patients	Intervention group participants experienced increased self-efficacy and independence, greater emotional safety, and maintenance of motivation. Adherence rate for this	Small sample size (<30), elderly patients (σ=55.2). Two-year pilot study, (N=10)

chronic obstructive pulmonary disease: a mixed methods study [18]		telerehabilitation program was higher than that reported by the World Health Organization for long-term therapy in chronic diseases.	
The effect of interactive Web-based monitoring on breastfeeding exclusivity, intensity, and duration in healthy, term infants after hospital discharge [19]	Web-based monitoring on breastfeeding exclusivity, intensity, and duration	No difference in postpartum depression between intervention and control groups (both decreased and were equally as effective). Effects of exclusivity and intensity were higher with intervention group, and effects on duration were equally effective in both groups.	The study underrepresented Hispanic and Asian populations. Randomized controlled trial (RCT), control (N=57), intervention (N=49).
A randomized controlled trial of personalized text message reminders to promote medication adherence among HIV-positive adolescents and young adults [20]	Personalized text message reminders to promote medication adherence among human immunodeficiency virus–positive adolescents and young adults	The intervention group demonstrated increased medication adherence and demonstrated durability of adherence post intervention.	Convenience sample of one geographical area, participants received payment. RCT, control (N=55), intervention (N=54).
A telehealth behavioral coaching intervention for	Telehealth program for depressive symptoms	Depressive symptoms, upset following disruptive behaviors, and negative mood states were statistically lower in the	Small sample size (<30). True experiment, pre-post design,

neurocognitive disorder family carers [21]		behavioral coaching condition than in the basic education and support condition. Caregiving self-efficacy scores for obtaining respite and for managing patient behavioral disturbances were significantly higher in the coaching condition.	control (N=50), intervention (N=50).
Clinical decision support and palivizumab: a means to protect from respiratory syncytial virus [22]	Using clinical decision support systems (CDSSs) to determine when palivizumab (expensive treatment) should be administered (under a complicated condition list).	A quality improvement initiative supported by clinical decision support (CDS) and workflow tools integrated in the electronic health record (EHR) improved recognition of eligibility and may have increased palivizumab administration rates; the palivizumab-focused group performed significantly better than a comprehensive intervention. Comprehensive intervention was associated with a trend toward increased missed doses that may be related to alert fatigue.	Coinventors involved in conducting the experiment. True experiment, control (N=162), intervention (N=194).

Scalable hospital at home with virtual physician visits: pilot study [23]	Hospital at Home (HaH) model with virtual physician visits	Safety and convenience were significantly higher rated than traditional method of treatment, Activities of daily living were significantly higher in the HaH group (5.81-5.39, *P*=.064), service utilization and mortality at 30 days was significantly lower in the HaH group (12-41, *P*<.001), number of specialty appointments was lower (0.34-1.50, *P*<.001), number of patients with hospital readmissions was lower (7-17, *P*=.072), cumulative service utilization and mortality at 90 days, total number of hospital readmissions (14-39, *P*<.002). Death was not statistically significant for 30 days or 90 days.	A large number of participants eligible for HaH refused. Quasi-experimental (nonrandomized), control (N=52), intervention (N=50).
Mortality benefits of antibiotic computerized decision support system: modifying effects of age [24]	CDSS for antibiotics	Patients aged ≤65 had greater mortality benefit (odds ratio, OR 0.45, 95% CI 0.20-1.00, *P*=.05) than patients that were older than 65 (OR 1.28, 95% CI 0.91-1.82, *P*=.16). No effect was observed on incidence of colostridium difficile (OR 1.02, 95% CI 0.34-3.01) and multidrug-resistant organism	Study does not include children. True experiment, control (N=99), intervention (N=99).

		(OR 1.06, 95% CI 0.42-2.71) infections. No increase in infection-related readmission (OR 1.16, 95% CI 0.48-2.79) was found in survivors. Receipt of CDSS-recommended antibiotics reduced mortality risk in patients aged 65 years or younger and did not increase the risk in older patients.	
Electronic stroke CarePath: integrated approach to stroke care [25]	Electronic stroke CarePath	There was a significant reduction in observed or expected inpatient mortality after implementation of the stroke CarePath in patients with ischemic stroke (OR 0.59, 95% CI 0.42-0.83), but not in the control patients with intracerebral hemorrhage (OR 0.90, 95% CI 0.59-1.38) or subarachnoid hemorrhage (OR 1.05, 95% CI 0.67-1.65). There was a significant increase in the proportion of ischemic stroke patients with length of stay (LOS) equal or less than expected LOS after CarePath implementation (*P*=.047), but there was no significant difference in patients with intracerebral hemorrhage	Significant technical challenges were experienced that resulted in delays in data availability. Quasi-experimental (nonrandomized), control (N=2852), intervention (N=1106).

		(*P*=.117) or subarachnoid hemorrhage (*P*=.943).	
Electronic ordering system improves postoperative pain management after total knee or hip arthroplasty [26]	Electronic ordering system	The time to initiation of analgesia from arrival in the post-anesthesia care unit (PACU) was significantly lower in the electronic group compared with the conventional group (mean=24.5, standard deviation [SD]=28.3 min vs mean=51.1, SD=26.2 min; *P*<.001), as were visual analog scale pain scores (mean=0.82, SD=1.08 vs mean=1.5, SD=1.52; *P*<.001) and the volume of patient-controlled epidural analgesia needed to control pain (mean=27.9, SD=20.2 mL vs mean=34.8, SD=20.3 mL; *P*=.001) at 4 hours postoperatively. PACU LOS and hospital LOS did not significantly differ in the two groups.	No bias identified. Quasi-experimental (nonrandomized), control (N=106), intervention (N=96).

The effectiveness of a patient-centered assessment with a solution-focused approach (DIALOG+) for patients with psychosis: a pragmatic cluster-randomized controlled trial in community care [27]	Patient-centered assessment with a solution-focused approach (DIALOG+) for patients with psychosis	Patients in the DIALOG+ arm had better subjective quality of life at 3, 6, and 2 months (*P*=.035, .058, and .014, respectively; Cohen *d*=0.29-0.34). The also had significantly fewer unmet needs at 3 and 6 months, fewer general psychopathological symptoms at all-time points, and better objective social outcomes at 12 months, with no significant differences in other outcomes.	No bias identified. Pragmatic, exploratory, parallel-group, cluster-RCT, control (N=85), intervention (N=94).
Reducing preconception risks among African American women with conversational agent technology [28]	Web-based preconception conversational agent system, “Gabby”	Emotional and mental health, risks triggered that were resolved/total no. of risks triggered (15/35, SD 43.0 vs 4/32, SD 13.0; *P*=.01). Health care, risks triggered that were resolved/total no. of risks triggered (8/24, SD 23.5 vs 7/64, SD 10.9); *P*=.09. Immunizations and vaccines, risks triggered that were resolved/total no. of risks triggered (25/80, SD 31.3 vs 21/111, SD 18.9; *P*=.05). Men and health care, risks triggered that were resolved/ total no. of risks triggered (7/64, SD 10.9 vs	African American women only. Results may not translate to the general population. RCT, control (N=31), intervention (N=46).

		14/39, SD 35.9; *P*=.01). Nutrition and activity, risks triggered that were resolved/total no. of risks triggered (101/292, SD 34 vs 67/302, SD 22.2; *P*=.01). Total risks triggered that were resolved/total no. of risks triggered (297/1067, SD 27.8 vs 224/1091, SD 20.5; *P*=.01)	
Feasibility and acute care utilization outcomes of a postacute transitional telemonitoring program for underserved chronic disease patients [29]	Telemonitoring of COPD and heart failure (HF) patients (for underserved population)	COPD patients who completed the program sustained clinically meaningful improvements in their health status. HF patients also improved their health through the program. Significantly reduction in the 30-day readmission rate.	Only for underserved population. Quasi-experimental (nonrandomized), controlHF (N=59), controlCOPD (N=174) interventionHF (N=59), interventionCOPD (N=58).
An electronic tool for the evaluation and treatment of sepsis in the ICU: a randomized controlled trial [30]	Electronic sepsis evaluation and management tool	There was no significant difference for mortality (14.3% vs 14.9%), ICU-free days (17 vs 19), or vasopressor-free days (22.2 vs 22.6) for the intervention compared with control.	No bias identified. Pragmatic randomized trial, control (N=189), intervention (N=218).
Effect of a computer-guided,	Computer-guided quality	Improved cardiovascular disease risk management,	No bias identified. Cluster-

quality improvement program for cardiovascular disease risk management in primary health care: the treatment of cardiovascular risk using electronic decision support cluster-randomized trial [31]	improvement intervention	patient in intervention were more likely to receive screening compared with control (62.8% vs 53.4%). No difference in prescription rates.	randomized trial, control (N=19,340), intervention (N=19,385).
Expert advice provided through telemedicine improves healing of chronic wounds: prospective cluster controlled study [32]	Telemedicine wound care	Significant improvement in wound healing compared with conventional practices (70% vs 45%).	No bias identified. Prospective cluster-controlled study, control (N=40), intervention (N=50).
Using electronic health record clinical decision support is associated with improved quality of care [33]	CDSS	Patients who visited clinics that were missing at least one of the CDS functions were more likely to have controlled blood pressure (86% vs 82%, OR 1.3; 95% CI 1.1-1.5) and more likely to not have adverse drug event visits (99.9% vs 99.8%; OR 3.0, 95% CI 1.3-7.3).	No bias identified. Retrospective review, quasi-experimental (nonrandomized), control (N=10,466), intervention (N=40,588).

Comparison of community health worker–led diabetes medication decision-making support for low-income Latino and African American adults with diabetes using e-health tools versus print materials: a randomized controlled trial [34]	Customized, interactive, Web-based tablet-computer delivered tools	Decrease in diabetes distress for the intervention group compared with control, but no difference in other outcomes.	No bias identified. Randomized clinical trial, control (N=95), intervention (N=93).
Integrating real-time clinical information to provide estimates of net clinical benefit of antithrombotic therapy for patients with atrial fibrillation [35]	Atrial fibrillation decision support tool	Significant gain in quality-adjusted life expectancy in 832/1876 patients (44.3%).	No bias identified. Retrospective cohort, (N=1876).

Implementation and evaluation of an integrated computerized asthma management system in a pediatric emergency department: a randomized clinical trial [36]	Computerized asthma management system in a pediatric emergency department (ED)	No difference in time to disposition decision. No change in hospital admission rate. No difference in ED length of stay.	Study of one center only. Randomized clinical trial, control (N=394), intervention (N=394).
The Utah Remote Monitoring Project: improving health care one patient at a time [37]	Telemonitoring	Reduced average A1C, declined systolic and diastolic blood pressure, decreased low-density lipoprotein (LDL) content	Used a convenience sample. Nonrandomized prospective observational preintervention-postintervention study, tracked A1C and blood pressure (BP) (N=89), tracked A1C only (N=12), tracked BP only (N=14).
Improving adherence for management of acute exacerbation of chronic	Electronic order sets for COPD management	Pneumococcal vaccination rate was higher (57% vs 45%, *P*=.02), there was an increase in long acting muscarinic antagonist usage during	No bias identified. Pre-post design, pre intervention (N=203), post intervention

obstructive pulmonary disease [38]		hospitalization (13% vs 25%, *P*=.002), and the median corticosteroid usage decreased by 49% in the postintervention group compared with the preintervention period.	(N=217).
Impact of nurse-led remote screening and prompting for evidence-based practices in the ICU [39]	Remote screening and prompting for evidence-based practices in the intensive care unit (ICU)	Patients in the postintervention period were more likely to receive daily sedation interruptions (interrater reliability [IRR]=1.57, 95% CI 1.45-1.71, *P*<.001) and daily spontaneous breathing trials (IRR=1.24, 95% CI 1.20-1.29). Patients in the postintervention period experienced shorter mean duration mechanical ventilation, ICU length of stay, and hospital length of stay.	No bias identified. Pre-post design, Pre intervention (N=4339), post intervention (N=8938).
Feasibility and effectiveness of an automated telehealth intervention to improve illness self-management in people with serious psychiatric and medical disorders [40]	Telehealth	Improvements in self-efficacy for managing depression and diastolic blood pressure. Reduction in urgent care and primary care visits	No bias identified. Single-arm pilot trial (N=70).

Effects of an individually tailored Web-based chronic pain management program on pain severity, psychological health, and functioning [41]	Web-based interventions	Decreased pain intensity and unpleasantness. Increase in quality of life.	No bias identified. Nonexperimental, pre-post design, (N=645).
Development and pilot testing of a mobile health solution for asthma self-management: asthma action plan smartphone application pilot study [42]	Mobile health technology	Patients demonstrated an improved asthma-related quality of life, the mean mini-Asthma Quality of Life Questionnaire score improved by 0.5 (*P*=.047). The Canadian Asthma Consensus Guidelines symptom benchmarks of asthma control reduced in the postintervention group.	Lack of control group, seasonal influence on asthma not controlled for as the study was only conducted during the summer months on 82% women, small sample size <30. (N=22)
The impact of EHR and HIE on reducing avoidable admissions: controlling main differential diagnoses [43]	Health information exchange	Fewer single day and 7-day readmission rates when medical history was viewed via EHR	No bias identified. Retrospective observational, (N=281,750).

Reliable individualized monitoring improves cholesterol control in kidney transplant recipients [44]	Automated previsit support from an EHR	Increased proportion of visits during which cholesterol monitoring was completed. Significant improvement with controlled LDL.	No bias identified. Interrupted time series cohort design, (N=62).
Web-based intervention to promote physical activity by sedentary older adults: randomized controlled trial [45]	Internet intervention with automated video and text for physical activity	Participants in the intervention group had significant gains in the level of activity compared with the control group	Large participant dropout. RCT, control (N=177), intervention (N=125).
Practice-based versus telemedicine-based collaborative care for depression in rural federally qualified health centers: a pragmatic randomized comparative effectiveness trial [46]	Telemedicine	Significant effects on response and admission rates. Greater reduction in severity over time.	No bias identified. Pragmatic randomized comparative effectiveness trial, control (N=151), intervention (N=132).

Internet-delivered cognitive-behavioral therapy v. conventional guided self-help for bulimia nervosa: long-term evaluation of a randomized controlled trial [47]	Internet-based guided self-help program for bulimia nervosa patients.	Intervention group had no significant difference in outcomes.	No bias identified. RCT, control (N=28), intervention (N=48).
Efficacy of a clinical decision-support system in an HIV practice: a randomized trial [48]	CDS	Participants in the intervention group had a higher average increase in cluster of differentiation 4 count compared with the control group (5.3 vs 3.2)	No bias identified. RCT, control (N=505), intervention (N=506).
Hospital implementation of health information technology and quality of care: are they related? [49]	Health information technology	Lower mortality, higher patient satisfaction, and higher assessments of quality of patient care.	On the basis of quality manager's survey response, (N=470).
How to improve drug dosing for patients with renal impairment in primary care—a cluster-randomized controlled trial [50]	Software program	Reduced inappropriately high doses of renally excreted medications compared with the control group (19.2% vs 34.5%)	No bias identified. Cluster-RCT, control (N=206), intervention (N=198).

The effects of combining Web-based eHealth with telephone nurse case management for pediatric asthma control: a randomized controlled trial [51]	Electronic health for kids with asthma	Asthma control as measured by Asthma Control Questionnaire improved significantly for the intervention group but not the control group (−0.42, −0.11)	No bias identified. RCT, control (N=127), intervention (N=132).

15. Müller KI, Alstadhaug KB, Bekkelund SI. A randomized trial of telemedicine efficacy and safety for nonacute headaches. *Neurology*. 2017 Jun 14:10-212. PMID: 28615434

16. Schulz-Heik RJ, Meyer H, Mahoney L, Stanton MV, Cho RH, Moore-Downing DP, Avery TJ, Lazzeroni LC, Varni JM, Collery LM, Bayley PJ. Results from a clinical yoga program for veterans: yoga via telehealth provides comparable satisfaction and health improvements to in-person yoga. *BMC complementary and alternative medicine*. 2017 Apr 4;17(1):198. PMID: 28376861

17. Feinberg AE, Elnahas A, Bashir S, Cleghorn MC, Quereshy FA. Comparison of robotic and laparoscopic colorectal resections with respect to 30-day perioperative morbidity. *Canadian Journal of Surgery*. 2016 Aug;59(4):262. PMID: 27240135

18. Hoaas H, Andreassen HK, Lien LA, Hjalmarsen A, Zanaboni P. Adherence and factors affecting satisfaction in long-term telerehabilitation for patients with chronic obstructive pulmonary disease: a mixed methods study. *BMC medical informatics and decision making*. 2016 Feb 25;16(1):26. PMID: 26911326

19. Ahmed AH, Roumani AM, Szucs K, Zhang L, King D. The effect of interactive web-based monitoring on breastfeeding exclusivity, intensity, and duration in healthy, term infants after hospital discharge. *Journal of Obstetric, Gynecologic & Neonatal Nursing*. 2016 Apr 30;45(2):143-54. PMID: 26779838

20. Garofalo R, Kuhns LM, Hotton A, Johnson A, Muldoon A, Rice D. A randomized controlled trial of personalized text message reminders to promote medication adherence among HIV-positive adolescents and young adults. *AIDS and Behavior*. 2016 May 1;20(5):1049-59. PMID: 26362167

21. Steffen AM, Gant JR. A telehealth behavioral coaching intervention for neurocognitive disorder family carers. *International journal of geriatric psychiatry*. 2016 Feb 1;31(2):195-203. PMID: 26077904

22. Utidjian LH, Hogan A, Michel J, Localio AR, Karavite D, Song L, Ramos MJ, Fiks AG, Lorch S, Grundmeier RW. Clinical decision support and palivizumab: a means to protect from respiratory syncytial virus. *Applied clinical informatics*. 2015 Dec 30;6(4):769. PMID: 26767069

23. Summerfelt WT, Sulo S, Robinson A, Chess D, Catanzano K. Scalable hospital at home with virtual physician visits: pilot study. *The American journal of managed care*. 2015 Oct;21(10):675-84. PMID: 26633092

24. Chow AL, Lye DC, Arah OA. Mortality benefits of antibiotic computerised decision support system: modifying effects of age. *Scientific reports*. 2015 Nov 30;5:srep17346. PMID: 26617195

25. Katzan IL, Fan Y, Speck M, Morton J, Fromwiller L, Urchek J, Uchino K, Griffith SD, Modic M. Electronic Stroke CarePath. Circulation: *Cardiovascular Quality and Outcomes*. 2015 Oct 1;8(6 suppl 3):S179-89. PMID: 26515207

26. Urban MK, Chiu T, Wolfe S, Magid S. Electronic Ordering System Improves Postoperative Pain Management after Total Knee or Hip Arthroplasty. *Applied clinical informatics*. 2015;6(3):591. PMID: 26448800

27. Priebe S, Kelley L, Omer S, Golden E, Walsh S, Khanom H, Kingdon D, Rutterford C, McCrone P, McCabe R. The effectiveness of a patient-centred assessment with a solution-focused approach (DIALOG+) for patients with psychosis: a pragmatic cluster-randomised controlled trial in community care. *Psychotherapy and psychosomatics*. 2015;84(5):304-13. PMID: 26278784

28. Bickmore, JB, Hempstead M, Yinusa-Nyahkoon L, Sadikova E, Mitchell S, Gardiner P, Adigun F, Penti B, Schulman D, Damus K. Reducing preconception risks among African American women with conversational agent technology. *The Journal of the American Board of Family Medicine*. 2015 Jul 1;28(4):441-51. PMID: 26152434

29. Davis C, Bender M, Smith T, Broad J. Feasibility and acute care utilization outcomes of a post-acute transitional telemonitoring program for underserved chronic disease patients. *Telemedicine and e-Health*. 2015 Sep 1;21(9):705-13. PMID: 25955129

30. Semler MW, Weavind L, Hooper MH, Rice TW, Gowda SS, Nadas A, Song Y, Martin JB, Bernard GR, Wheeler AP. An electronic tool for the evaluation and treatment of sepsis in the ICU: a randomized controlled trial. *Critical care medicine*. 2015 Aug;43(8):1595. PMID: 25867906

31. Peiris D, Usherwood T, Panaretto K, Harris M, Hunt J, Redfern J, Zwar N, Colagiuri S, Hayman N, Lo S, Patel B. Effect of a computer-guided, quality improvement program for

cardiovascular disease risk management in primary health care. *Circulation: Cardiovascular Quality and Outcomes*. 2015 Jan 1;8(1):87-95. PMID: 25587090

32. Zarchi K, Haugaard VB, Dufour DN, Jemec GB. Expert advice provided through telemedicine improves healing of chronic wounds: prospective cluster controlled study. *The Journal of investigative dermatology*. 2015 Mar 1;135(3):895. PMID: 25290685

33. Mishuris RG, Linder JA, Bates DW, Bitton A. Using electronic health record clinical decision support is associated with improved quality of care. *Am J Manag Care*. 2014 Oct 1;20(10):e445-52. PMID: 25414982

34. Heisler M, Choi H, Palmisano G, Mase R, Richardson C, Fagerlin A, Montori VM, Spencer M, An LC. Comparison of community health worker-led diabetes medication decision-making support for low-income Latino and African American adults with diabetes using e-health tools versus print materials: a randomized, controlled trial. *Annals of internal medicine*. 2014 Nov 18;161(10_Supplement):S13-22. PMID: 25402398

35. Eckman MH, Wise RE, Speer B, Sullivan M, Walker N, Lip GY, Kissela B, Flaherty ML, Kleindorfer D, Khan F, Kues J. Integrating real-time clinical information to provide estimates of net clinical benefit of antithrombotic therapy for patients with atrial fibrillation. *Circulation: Cardiovascular Quality and Outcomes*. 2014 Sep 1;7(5):680-6. PMID: 25205788

36. Dexheimer JW, Abramo TJ, Arnold DH, Johnson K, Shyr Y, Ye F, Fan KH, Patel N, Aronsky D. Implementation and evaluation of an integrated computerized asthma management system in a pediatric emergency department: a randomized clinical trial. *International journal of medical informatics*. 2014 Nov 30;83(11):805-13. PMID: 25174321

37. Shane-McWhorter L, Lenert L, Petersen M, Woolsey S, McAdam-Marx C, Coursey JM, Whittaker TC, Hyer C, LaMarche D, Carroll P, Chuy L. The Utah Remote Monitoring Project: improving health care one patient at a time. *Diabetes technology & therapeutics*. 2014 Oct 1;16(10):653-60. PMID: 24991923

38. Sonstein L, Clark C, Seidensticker S, Zeng L, Sharma G. Improving adherence for management of acute exacerbation of chronic obstructive pulmonary disease. *The American journal of medicine*. 2014 Nov 30;127(11):1097-104. PMID: 24927911

39. Kahn JM, Gunn SR, Lorenz HL, Alvarez J, Angus DC. Impact of nurse-led remote screening and prompting for evidence-based practices in the ICU. *Critical care medicine*. 2014 Apr;42(4):896. PMID: 24201176

40. Pratt SI, Bartels SJ, Mueser KT, Naslund JA, Wolfe R, Pixley HS, Josephson L. Feasibility and effectiveness of an automated telehealth intervention to improve illness self-management in people with serious psychiatric and medical disorders. *Psychiatric rehabilitation journal*. 2013 Dec;36(4):297. PMID: 24320837

41. Nevedal DC, Wang C, Oberleitner L, Schwartz S, Williams AM. Effects of an individually tailored web-based chronic pain management program on pain severity, psychological health, and functioning. *Journal of medical Internet research*. 2013 Sep;15(9). PMID: 24067267

42. Licskai CJ, Sands TW, Ferrone M. Development and pilot testing of a mobile health solution for asthma self-management: asthma action plan smartphone application pilot study. *Canadian respiratory journal*. 2013;20(4):301-6. PMID: 23936890

43. Ben-Assuli O, Shabtai I, Leshno M. The impact of EHR and HIE on reducing avoidable admissions: controlling main differential diagnoses. *BMC medical informatics and decision making*. 2013 Apr 17;13(1):49. PMID: 23594488

44. Hooper DK, Kirby CL, Margolis PA, Goebel J. Reliable individualized monitoring improves cholesterol control in kidney transplant recipients. *Pediatrics*. 2013 Mar 1:peds-2012. PMID: 23478865

45. Irvine AB, Gelatt VA, Seeley JR, Macfarlane P, Gau JM. Web-based intervention to promote physical activity by sedentary older adults: randomized controlled trial. *Journal of Medical Internet Research*. 2013 Feb;15(2). PMID: 23470322

46. Fortney JC, Pyne JM, Mouden SB, Mittal D, Hudson TJ, Schroeder GW, Williams DK, Bynum CA, Mattox R, Rost KM. Practice-based versus telemedicine-based collaborative care for depression in rural federally qualified health centers: a pragmatic randomized comparative effectiveness trial. *American Journal of Psychiatry*. 2013 Apr;170(4):414-25. PMID: 23429924

47. Wagner G, Penelo E, Wanner C, Gwinner P, Trofaier ML, Imgart H, Waldherr K, Wöber-Bingöl Ç, Karwautz AF. Internet-delivered cognitive–behavioural therapy v. conventional guided self-help for bulimia nervosa: long-term evaluation of a randomised controlled trial. *The British Journal of Psychiatry*. 2012 Nov 30:bjp-p. PMID: 23222037

48. Robbins GK, Lester W, Johnson KL, Chang Y, Estey G, Surrao D, Zachary K, Lammert SM, Chueh HC, Meigs JB, Freedberg KA. Efficacy of a Clinical Decision-Support System in an HIV Practice: A Randomized Trial. *Annals of internal medicine*. 2012 Dec 4;157(11):757-66. PMID: 23208165

49. Restuccia JD, Cohen AB, Horwitt JN, Shwartz M. Hospital implementation of health information technology and quality of care: are they related?. *BMC medical informatics and decision making*. 2012 Sep 27;12(1):109. PMID: 23016699

50. Erler A, Beyer M, Petersen JJ, Saal K, Rath T, Rochon J, Haefeli WE, Gerlach FM. How to improve drug dosing for patients with renal impairment in primary care-a cluster-randomized controlled trial. *BMC family practice*. 2012 Sep 6;13(1):91. PMID: 22953792

51. Gustafson D, Wise M, Bhattacharya A, Pulvermacher A, Shanovich K, Phillips B, Lehman E, Chinchilli V, Hawkins R, Kim JS. The effects of combining Web-based eHealth with telephone nurse case management for pediatric asthma control: a randomized controlled trial. *Journal of medical Internet research*. 2012 Jul;14(4). PMID: 22835804
